# Evaluation of the Performance and Safety of a New Micro‐Needle Technology in Comparison With the Classic Needle on the Antiaging Effects of a Biorevitalizing Solution: A Randomized Split Face/Neck Study

**DOI:** 10.1111/jocd.16547

**Published:** 2024-10-02

**Authors:** Andreea Boca, Ferial Fanian, Riekie Smit, Alessio Redaelli, Ranesha Goorochurn, Hanane Issa, Natalia Sukmanskaya, Valérie Philippon, Roberto Dell’ Avanzato

**Affiliations:** ^1^ Private Clinic Cluj Romania; ^2^ Scientific Department FILLMED Laboratories Paris France; ^3^ Private Clinic Pertoria South Africa; ^4^ Genova University Milan Italy; ^5^ Dermo Dev Partners Paris France; ^6^ FILLMED Laboratories Paris France; ^7^ Private Clinic Paris France; ^8^ Private Clinic Milan Italy

**Keywords:** HA, micro‐needle, multiple intradermal injection, new technology, skin aging, skin biorevitalization

## Abstract

**Background:**

Skin biorevitalization involves multiple intradermal injections to enhance skin quality, but precise dermal targeting can be challenging due to variations in skin thickness smaller, less painful needles with fewer skin reactions are attractive options.

**Aims:**

This study evaluates a new Micro‐Needle device's performance and safety in comparison with the classic needle used in skin biorevitalization.

**Patients/Methods:**

Subjects with facial and neck skin aging were enrolled. Safety outcomes, including immediate and local tolerability, were assessed. Performance outcomes measured skin radiance, wrinkles and photoaging grade, hydration, subepidermal low echogenic band, dermis thickness, and skin elasticity. Both subjects and investigators recorded Global Aesthetic Improvement Scale scores.

**Results:**

Micro‐Needle injections demonstrated superior performance compared to the classic needle, influenced by the specific skin zones and thickness. Micro‐Needle was superior for skin wrinkles at D49 for periorbital zone and nasolabial folds by −14.5% (*p* = 0.01) and −15% (*p* = 0.004), respectively, and for neck by 9.6% (*p* = 0.0008). The Nanosoft device showed a faster improvement for skin hydration at D42 for the cheek zone (*p* = 0.04) and at D75 for the neck area (*p* = 0.01); and for skin radiance at D75 (*p* = 0.03) and at D120 (*p* = 0.0098). Ex vivo studies confirmed the Micro‐Needle's accuracy in product placement in the dermis. Adverse events were milder with Micro‐Needle and no serious adverse events occurred.

**Conclusions:**

Both needles significantly improved skin quality, but Micro‐Needle enhanced the outcomes of skin biorevitalization procedures, particularly in terms of skin wrinkle reduction, elasticity, and overall skin hydration.

## 1 Introduction

The aging perception by patients is the most frequent reason for a medical consultation. Both intrinsic and extrinsic factors, participate in the aging process of the skin. In addition, photo‐exposed areas, such as the face or hands, are subject to the cumulative effect of both chronological aging and environmental factors [1, 2]. Face aging of the superficial plane (skin) and that of the support structures (fat, muscles, and bones) are distinguished. At the level of the skin a reduction in cell renewal, dehydration, loss of radiance, elasticity, firmness, and the appearance of fine lines and wrinkles are observed. Structural aging (deep planes) causes atrophy of the bone structures and melting of the fats associated with a displacement of the latter toward the bottom of the face (gravity). This results in sagging skin in the middle and lower third of the face (cheeks, oval), accompanied by wrinkles and furrows increasingly marked.

Nonsurgical cosmetic solutions have enabled to offer simple and efficient treatments to patients wishing to slow down facial aging. However, as the barrier properties of skin limit the transport of molecules, various chemical and physical permeation enhancement techniques have been deployed for the delivery of active molecules through skin [3, 4, 5, 6]. Among the most recent administration techniques and attractive treatment option, the application of microneedle‐based devices is a favorable drug administration approach for skin rejuvenation [7, 8].

In this clinical trial, we use NCTF 135HA (FILLMED Laboratories, Paris, France) as a skin rejuvenation strategy thanks to its effects previously demonstrated on tissue filling of fine lines with hyaluronic acid and restructuring of the extracellular matrix by maintaining the hydration of the skin and its biochemical and biological architecture [9, 10]. NCTF 135HA is an antiaging biorevitalization solution containing 59 nutritive ingredients and 5 mg/mL of non–cross‐linked hyaluronic acid.

The administration of NCTF 135HA product, mesotherapy product, with the indication of biorevitalization is defined as a minimally invasive cosmetic medical treatment, which involves the intradermal injection directly in the zone to treat of active substances, to achieve the best efficiency. In order to penetrate the epidermis and upper dermal layer of the skin, Micro‐Needle devices can be presented either in a single needle—with length range of 4–6 mm for Mesoneedle and 1–2 mm for Mesogun—or an array of micron‐sized needles—with length range of 0.25–2 for Needle Pen and 0.5–1.5 mm for DermaRoller.

However, the longer needles are the more adverse effects can be expected such as erythema, edema, hyperpigmentation, and scarring on the skin [8]. The literature showed that more studies are required to assess the safety profile of biorevitalization to manage and minimize the risk of potential adverse reactions, mostly for isolated cases [11, 12].

Therefore, in this study, our purpose was to determine the performance and safety of a new Micro‐Needle device based on vaccine delivery clinically approved system (Nanopass Technologies) [3, 13] in the aim to better manage the risk of adverse effects linked to intradermal injections.

## 2 Materials and Methods

### 2.1 Subject Selection

The inclusion criteria were the male or female subjects older than 19 years old with a Fitzpatrick phototype of I to IV and a photoaging grade of 2 or 3 on Glogau scale; a Lemperle wrinkle score of 2–4 for periorbital lines and a Bazin neck wrinkle score of 2–4. Female subject accepted to do a pregnancy test. The noninclusion criteria included the participants with any allergy to the study product, history of dermal fillers during last 1 year, history of keloid scars, facial herpes, autoimmune disease, coagulation disorders, any acute inflammation/infection or any other medication, condition or disease which may interfere the results by investigator decision.

### 2.2 Objectives

The main objective was to demonstrate objectively the difference between the efficacy and safety of hyaluronic acid‐based solution injected bilaterally and randomly on the face and neck treated with Micro‐Needle technology (Nanosoft, Micro‐Needle, Nanopass Technologies Ltd., Israel) versus the other side treated with classic needle from D0 to D75 (30 days after the third and last treatment).

### 2.3 Study Design

The study adapted with the declaration the Helsinki with the authorization of the local ethical committee of Medical University of “Iuliu Haţieganu” under registration number 2/1101.2019. All participants signed a written consent form, accepting not modifying their lifestyle and avoiding the sun exposure during the whole study. This study is a randomized, comparative, prospective monocenter study for 120 days.

The protocol consisted of three injection sessions, 3 weeks apart at Day 0, D21, D42 and three follow‐up sessions at D49 (7 days after the last injection session), D75 and D120.

The physician disinfected the treated area with chlorhexidine and injected the biorevitalizing solution (NCTF 135HA) which was prepared in a 3 mL sterile syringe through a 32G × 4 mm classic needle on one side or through a Micro‐Needle (Nanosoft) for the other side in a randomized way. The injection volume for each zone was as follows: 2 vials of 3 mL for whole face (3 mL per side) and 1 vial of 3 mL for the neck (1.5 mL per side). The treatment based on the multiple intradermal injections on whole face and neck spaced every 1–1.5 cm with a quantity of 0.05 mL on each point to produce a visible papula.

### 2.4 Investigated Products

#### 2.4.1 Micro‐Needle Nanosoft

**FIGURE 1:**
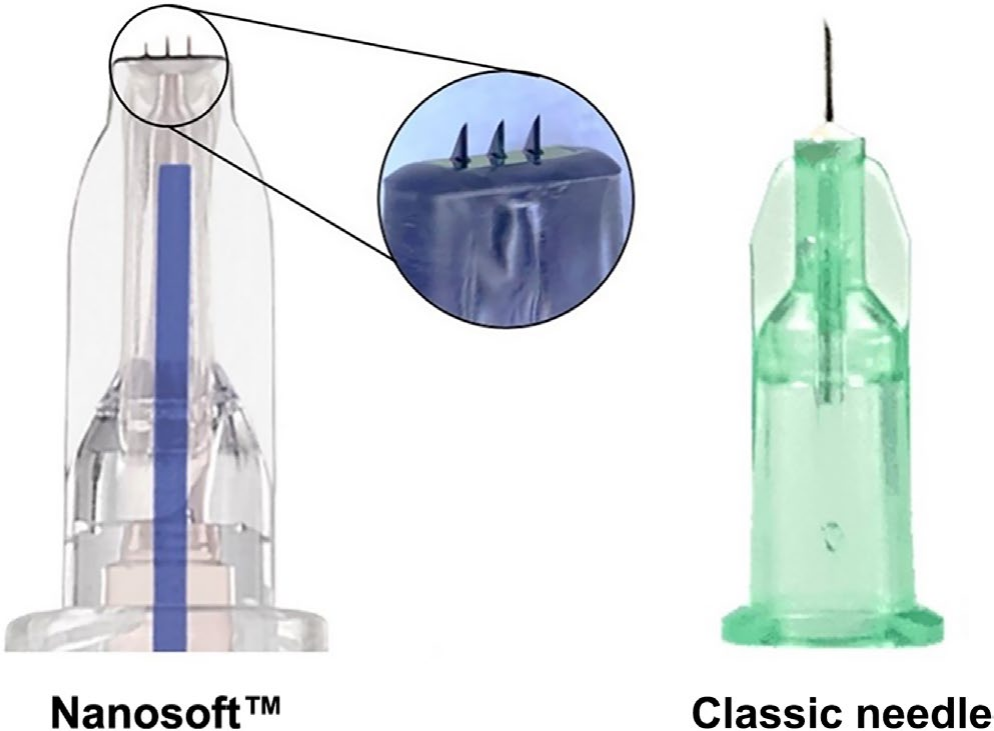
The injector devices, Nanosoft with three silicon needles of 0.6 mm and classic needle, 32 gage with 4 mm length.

A latex‐free CE marked Micro‐Needle technology with three silicon‐based needles of 0.6 mm (Figure 1). The Micro‐Needle enables to control intradermal delivery in any procedure which requires administration of substances to the dermal compartment. This device was used previously for vaccination and recently is introduced for the first time in aesthetic indications by FILLMED Laboratories.

#### 2.4.2 Classic Needle

We used a 32‐gage 4‐mm (32G × 3/16″) needle, TSK Laboratory, Japan, EMERGO EUROPE (Figure 1).

#### 2.4.3 Biorevitalizing Solution

NCTF 135HA (FILLMED Laboratories, France) is a 3‐mL vial containing 5 mg/mL of non–cross‐linked sodium hyaluronate and a polyrevitalizing solution (described in Table 1).

**TABLE 1:** Complete ingredients of NCTF135HA.

Compound class	Components
Vitamins total: 12	Ascorbic acid (vit. C), biotin (vit. B8), pantothenic acid (vit. B5), folic acid (vit. B9), inositol (vit. I), nicotinamide (vit. B3), pyridoxine (vit. B6), riboflavin (vit. B2), thiamine (vit. B1), tocopherol (vit. E), retinol (vit. A), vit. B12
Minerals total: 6	Calcium chloride, potassium chloride, magnesium sulfate, sodium acetate, sodium chloride, sodium dihydrogenophosphate
Nucleosides total: 5	Deoxyadenosine, deoxycytidine, deoxyguanosine, deoxythymidine, 5‐methyl‐2′‐deoxycytidine
Amino acids total: 24	α‐Aminobutyric acid, alanine, arginine, asparagine, aspartic acid, cystine, glutamine, glutamic acid, glycine, histidine, hydroxyproline, isoleucine, leucine, lysine, methionine, ornithine, phenylalanine, proline, serine, taurine, threonine, tryptophane, tyrosine, valine
Coenzymes total: 6	TPP (Cocarboxylase), CoA (coenzyme A), FAD (flavine adenine dinucleotide), NAD (nicotinamide adenine dinucleotide), NADP (nicotinamide adenine dinucleotide phosphate), UTP (uridine triphosphate)
Other compounds total: 6	Glutathione, polysorbate 80, glucuronic acid, glucuronic acid lactone, glucosamine, dextrose anhydrous

### 2.5 Pre‐Clinical Evaluation of Nanosoft Versus Classic Needle

**FIGURE 2:**
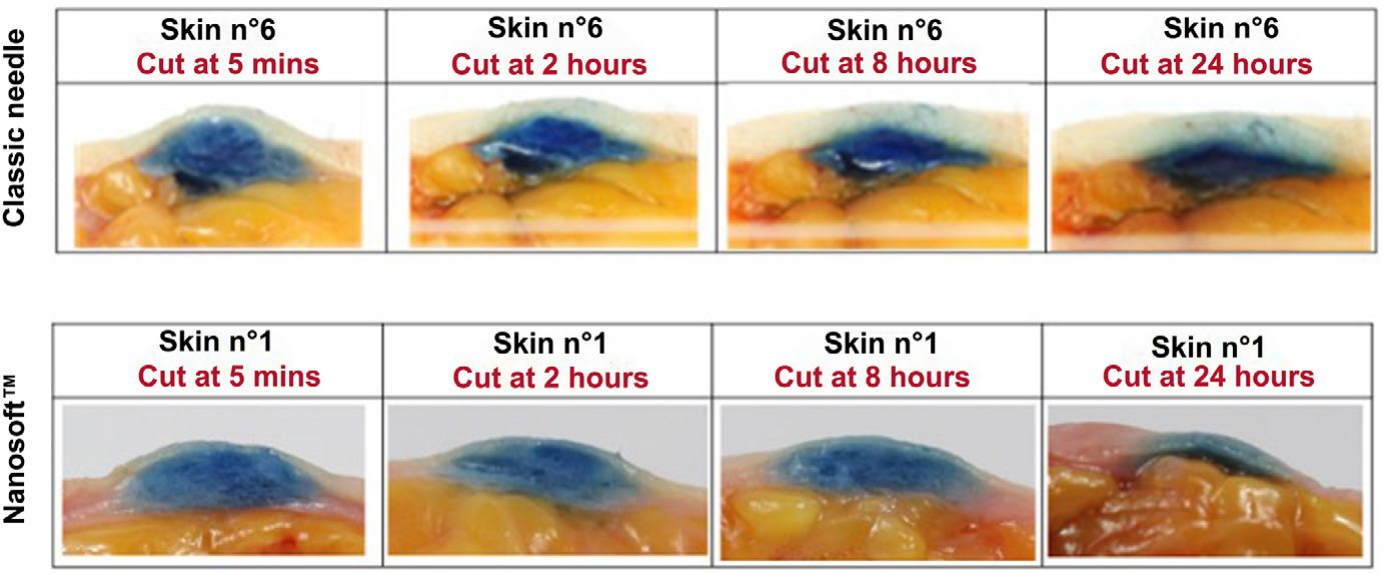
Monitoring over time of papules formed by injection of colored NCTF 135 HA, by Nanosoft and by a 32‐gage 4 mm classic needle. The skin was cut immediately, 2, 8, and 24 h after the injection.

In an ex vivo study performed internally with a colored NCTF 135HA, both Nanosoft and 32G × 4 mm classic needle were evaluated for their capability to produce a papula in the dermis and the duration of its persistence. After the injection the explant was cut at 2, 6, 8, and 24 h (Figure 2). The results show that regardless of the injection method, the papules became flattened till 24 h. However, a visible diffusion of the colored injected product in the hypodermis is observed for the classic needle while the product remained intact in the dermis with no diffusion in the hypodermis for the skin injected by the Nanosoft (Figure 2). This diffusion could waste the product form its main target which is the superficial and deep dermis.

### 2.6 Evaluation Methods

#### 2.6.1 Antiaging Performance Measures

The clinical assessment was performed by a visual scoring system regarding the skin radiance, skin wrinkles in different zones (face and neck), and the photoaging Glogau Scale (Table 2).

**TABLE 2:** Summary of evaluation methods.

Parameters	Scoring system	Zone(s)	Scales
Skin radiance	Skin radiance clinical scoring	Face	0: Very dull skin 1: Dull skin, lacking radiance 2: Slightly radiant skin 3: Radiant skin 4: Very radiant skin
Skin wrinkles	Lemperle score [14]	Nasolabial periorbital cheek	0: No wrinkle 1: Very shallow, still visible wrinkle 2: Shallow wrinkles 3: Moderately deep wrinkles 4: Deep wrinkles, well defined edges 5: Very deep wrinkles, redundant folds
	Bazin photographic visual score [15]	Neck	0: No wrinkle 1: Very shallow, still visible wrinkle 2: Shallow wrinkles 3: Slight wrinkles 4: Mild wrinkles 5: Deep wrinkles 6: Very deep wrinkles
Global photoaging	Glogau scale [16]	Face	I: Mild—no wrinkles, early photoaging II: Moderate—wrinkles in motion, early to moderate photoaging III: Advanced—wrinkles at rest, advanced photoaging IV: Severe—only wrinkles, severe photoaging

Parameters	Technique/device	Assessed area	Values
Skin hydration	Moisturemeter EpidD, Delfin Technology	–Face (cheeks) –Neck	Hydration level of epidermis (percentage)
Subepidermal Low Echogenic Band (SLEB) [17]	High Frequency Ultrasound imaging (DermaLab, Cortex Technology)	–Face (cheeks) –Neck	SLEB index in μm
Dermis thickness	High Frequency Ultrasound imaging (DermaLab, Cortex Technology)	–Face (periorbital zone and cheeks) –Neck	Thickness in μm
Skin Elasticity	DermaLab, Cortex Technology	–Face (periorbital zone and cheeks) –Neck	Overall elasticity (VE index) in MPa/mms

The instrumental assessment was carried out using various techniques detailed in Table 2.

The satisfaction rate was assessed by seven grades Global Aesthetic Improvement Scale (GAIS) from −3 to +3 evaluated by the investigators and also the subjects with following description (very much improved [+3], much improved [+2], improved [+1], no change [0], worse [−1], much worse [−2], and very much worse [−3]).

#### 2.6.2 Safety

Safety analysis includes all subjects who received at least one injection session with one of the devices under study. Immediate tolerance was assessed by measuring the pain based on an analog 10 grades visual scale from 0 for no pain to 10 for very intense pain. All local adverse events associated with the injection were recorded as well. They were scored by the investigator at each visit based on a 0–3 scale from absent to very severe from the first injection until the end of the study. These expected local adverse events are including erythema, ecchymosis, hematoma, edema, dyschromia, nodule/papule, and pruritus. In parallel, the patients recorded any local or systemic reactions or disorders on a daily log which was evaluated in each time point by investigator and recorded them in the CRF.

### 2.7 Statistical Methodology

The main criterion for this study is based on 5‐point scale clinical scoring (either Lemperle clinical scoring for face or Bazin clinical scoring for neck). For these scales, a decrease of at least one point demonstrates an apparent aesthetic evolution which indicated the success rate. Assuming a standard deviation of the after‐before differences equal to 2, then 35 patients per group are required to have a 90% chance of detecting a difference between means of 1.0 with a significance level (alpha) of 5% (calculated with the sample size for paired *t*‐test [one‐tailed]). As this study is a split face/neck study, the minimum number is 35 subjects. Considering the drop off rate of patients, a total 40 subjects were included. Statistical analysis performed with Statistica Version 12, Graphpad Instat and Excel 2016. Descriptive statistics are provided for each parameter (i.e., number of observations, mean, standard‐deviation [SD], minimum, maximum, median, 95% confidence interval).

The repartition of the sample size is provided for the evolution of the clinical scores in the different classes of scores under the form of n/percentage. Analyses is performed per area and per treatment.

The statistical significance of the evolution of the score between D0 and other time points was checked by a paired‐series Student's *t*‐test or its nonparametric equivalent and the superiority of the treatment with Micro‐Needle was tested by comparing evolution observed with both treatments (one‐sided Student's *t*‐test or Wilcoxon test on the deltas *D0‐Dx*).

## 3 Results

### 3.1 Study Population

Forty healthy subjects between 32 and 69 years old (mean age: 46.9 years) were enrolled in the study in Cluj, Romania including 5 male and 35 female volunteers. Twenty‐one patients were included in March 2019 and 20 patients were included in August 2020; one subject voluntarily stopped the study just after one injection. Analyses were thus performed on population per protocol (PP): *N* = 40 subjects. Among them, only six had previously received aesthetic treatments (hyaluronic acid/fillers, botulinum toxin or biorevitalization) with an acceptable delay according to the noninclusion criteria.

### 3.2 Skin Radiance

**FIGURE 3:**
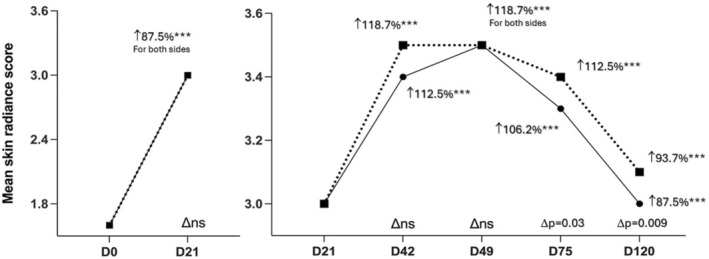
Mean skin radiance score from very dull skin (Grade 0) to very radiant skin (Grade 4). Significance value indicate ***p<0.001.

A significant improvement of skin radiance score was observed as early as 3 weeks after only one injection (D21). This result remains significant for all time points versus baseline till D120 for both face sides and devices (*p* < 0.0001 for all time points) (Table 3 and Figure 3). The difference between two devices is statistically significant at D75 (*p* = 0.03) and at D120 (*p* = 0.0098) in favor of Nanosoft.

**TABLE 3:** Clinical scoring of different time points for Micro‐Needle or classic needle.

	Classic needle			Micro‐Needle			
*n* = 40	Mean	SD	*p*	Mean	SD	*p*	∆*p*
Skin radiance grade							
D0	1.6	0.6	—	1.6	0.6	—	—
D21	3.0	0.6	<0.0001	3.0	0.6	<0.0001	ns
D42	3.4	0.5	<0.0001	3.5	0.5	<0.0001	ns
D49	3.5	0.6	<0.0001	3.5	0.6	<0.0001	ns
D75	3.3	0.6	<0.0001	3.4	0.6	<0.0001	0.0363
D120	3.0	0.3	<0.0001	3.1	0.5	<0.0001	0.0098
Glogau score							
D0	2.60	0.46	—	2.60	0.50	—	—
D120	2.20	0.60	<0.0001	2.10	0.50	<0.0001	ns
Skin wrinkles score							
Cheek zone							
D0	1.3	0.6	—	1.4	0.6	—	—
D49	0.6	0.5	<0.0001	0.5	0.5	<0.0001	ns
D75	0.7	0.6	<0.0001	0.6	0.5	<0.0001	0.0205
D120	0.8	0.6	<0.0001	0.7	0.5	<0.0001	0.0205
Periorbital zone							
D0	2.4	0.7	—	2.5	0.8	—	—
D49	1.5	1.2	<0.0001	1.2	1.1	<0.0001	0.0181
D75	1.7	1.0	<0.0001	1.5	1.1	<0.0001	0.0212
D120	1.8	1.0	<0.0001	1.5	1.0	<0.0001	0.0004
Nasolabial folds							
D0	2.0	0.9	—	2.0	1.0	—	—
D49	1.3	0.9	<0.0001	1.0	0.9	<0.0001	0.0041
D75	1.4	1.0	<0.0001	1.2	0.9	<0.0001	0.0178
D120	1.6	0.9	<0.0001	1.4	0.9	<0.0001	0.0286
Neck zone							
D0	2.7	0.7	—	2.8	0.9	—	—
D49	1.8	0.9	<0.0001	1.6	0.9	<0.0001	0.0008
D75	2.0	0.8	<0.0001	1.8	1.0	<0.0001	0.0002
D120	2.0	0.8	<0.0001	1.9	0.8	<0.0001	0.0017

	Classic needle			Micro‐Needle		
***n*** = 40	∆mean (%)			∆mean (%)		
	D49	D75	D120	D49	D75	D120
Cheek	−53.8	−46.2	−38.5	−64.3	−57.1	−50.0
Periorbital	−37.5	−30.0	−40.0	−52.0	−40.0	−40.0
Nasolabial	−35.0	−30.0	−20.0	−50.0	−40.0	−30.0
Neck	−33.3	−25.9	−25.9	−42.9	−35.7	−32.1

### 3.3 Photoaging Assessments

The data revealed a significant improvement of Glogau photoaging score after 4 months of treatment (D120) compared to the baseline (D0) for both treated sides (*p* < 0.0001 for all) (Table 3). This positive evolution allows a global photoaging improvement no matter the treatment options with no significant difference between both modes of injection. This observation is due to the direct effect of the biorevitalizing solution (NCTF 135HA) and not due to the injector.

### 3.4 Skin Wrinkles

**FIGURE 4:**
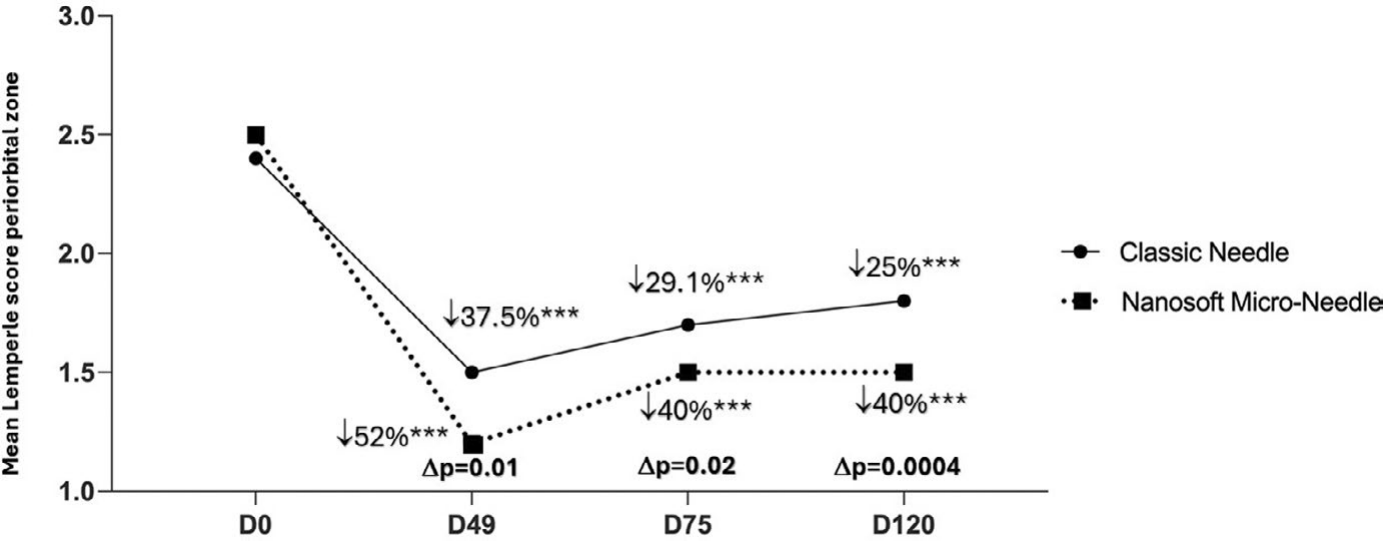
Mean Lemperle score on periorbital zone on different time points. Significance value indicate ***p<0.001.

**FIGURE 5:**
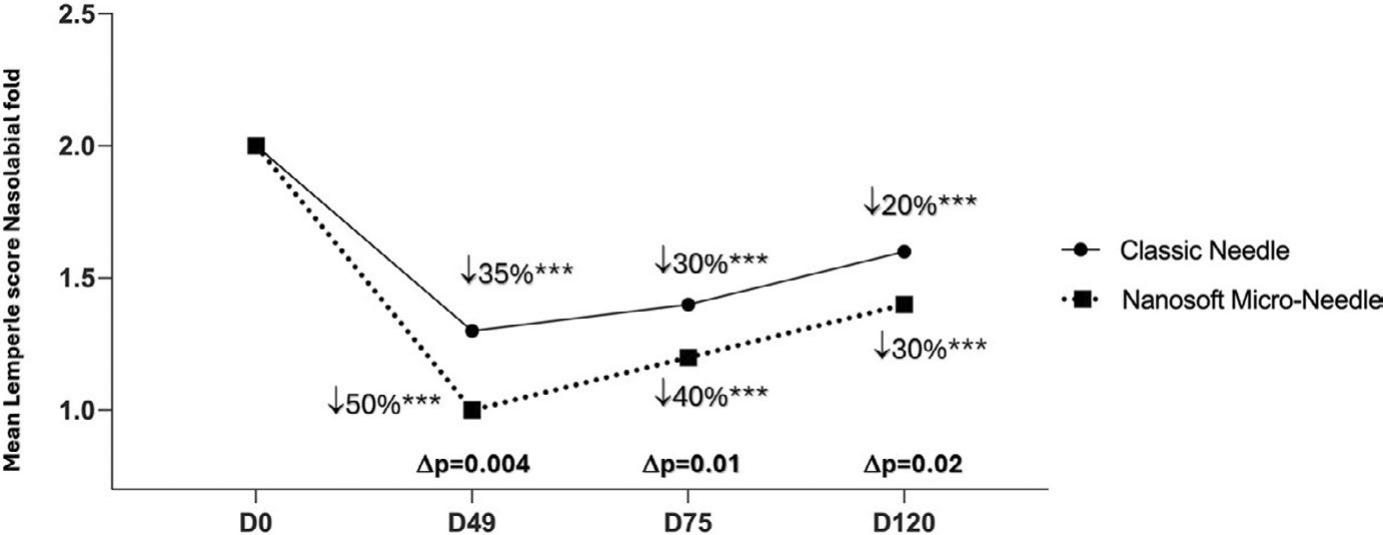
Mean Lemperle score on nasolabial fold on different time points. Significance value indicate ***p<0.001.

**FIGURE 6:**
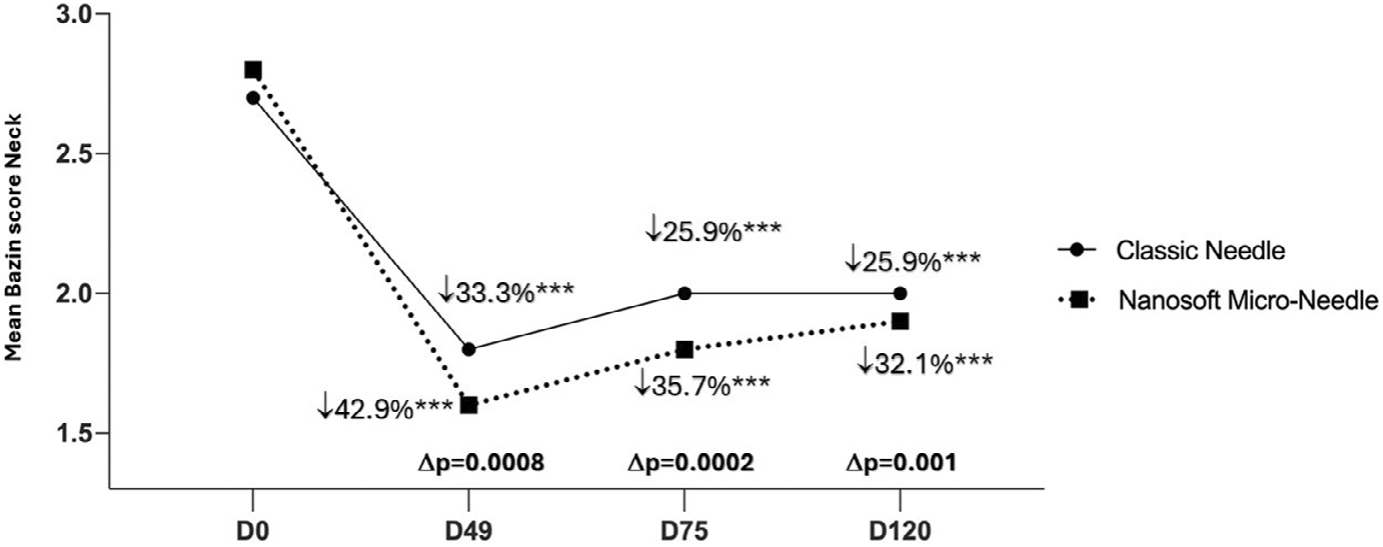
Mean Bazin score on neck on different time points. Significance value indicate ***p<0.001.

A significant improvement of skin wrinkles score was obtained for face on cheeks, periorbital area and nasolabial fold as well as on neck for all time points: at D49 (7 days after 3 injections), at D75 (1 month after 3 injections) and at D120 (2.5 months after 3 injections) versus baseline, for both face sides (*p* < 0.0001 for all) (Table 3). A statistical difference in favor of Micro‐Needle was reported for every time point and for all examined sites, except at D49 for the cheek area which still show a tendency difference in favor of Micro‐Needle (*p =* 0.054). The peak decrease of skin wrinkles score was obtained on the cheek at D49 compared to baseline, by 53.8% for classic needle versus 64.3% for microneedle side. The percentage of the evolution for other zones at the same time point (D49) was reported as: 52% versus 37.5% for periorbital zone (*p* = 0.02), 50% versus 35% for nasolabial fold (*p* = 0.01) and 42.9% versus 33.3% for neck wrinkles (*p* = 0.0002) (Table 3). Furthermore, the difference between the percentage of skin wrinkles with Micro‐Needle and classic needle showed that the highest diminution score difference was in favor of Micro‐Needle technique, for the periorbital by −14.5% (*p* = 0.01) (Figure 4), for nasolabial folds by −15% (*p* = 0.004) (Figure 5) and for neck by 9.6% (*p* = 0.0008) (Figure 6 and Table 3).

### 3.5 Skin Quality

The assessment of high‐frequency ultrasound and skin elasticity by DermaLab (Cortex Technology, Denmark) shows a significant improvement of dermis thickness and the overall elasticity versus baseline for both treated sides.

#### 3.5.1 Cheeks

**FIGURE 7:**
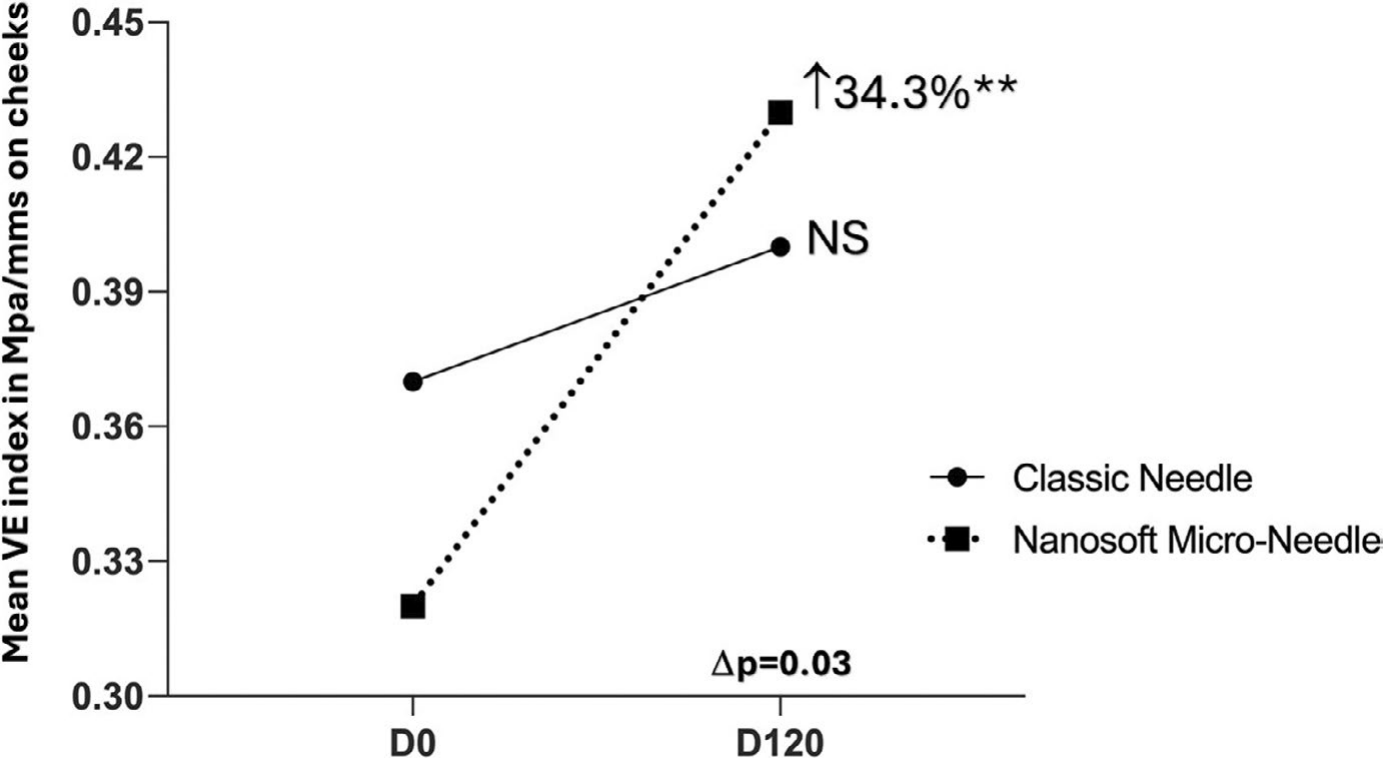
Mean overall elasticity by VE index (in MPa/mms) assessed on the cheek. Significance value indicate **p<0.01.

The cheek area also revealed a raise of the dermis thickness at D49 with Micro‐Needle technology (*p* < 0.02). Regarding skin elasticity, the side treated with Micro‐Needle device showed an improvement of the overall elasticity (mean VE index) for this zone with an increase of 34% at D120 compared to the baseline (*p* = 0.006) while the evolution on classic needle was non‐significant (Figure 7 and Table S1).

#### 3.5.2 Periorbital Area

The data obtained for the overall elasticity indicated a significant difference of evolution between treatments in favor to Micro‐Needle technology for periorbital zone after 4 months of treatment at D120 (*p* < 0.05) (Table S1).

#### 3.5.3 Neck

**FIGURE 8:**
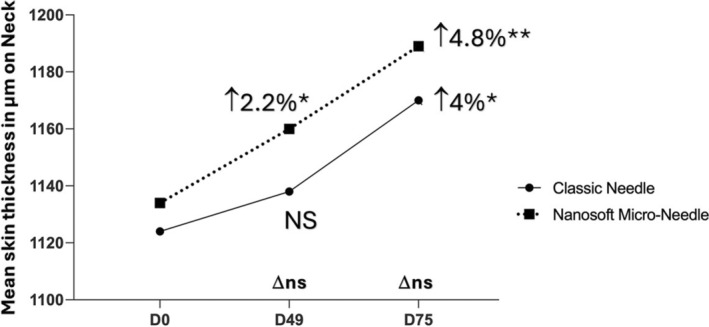
Mean dermis thickness in μm assessed on the neck zone on different time points. Significance value indicates *p<0.05, **p<0.01.

The neck zone presented a significant increase of the dermis thickness only for the side treated with the Micro‐Needle, 7 days and 30 days after the third and last treatment (*p* < 0.05 and *p* < 0.002, respectively); whereas the side treated by classic needle showed a slight increase of the dermis thickness only at 30 days after the third and last treatment (*p* < 0.05) (Figure 8 and Table S1).

### 3.6 Skin Hydration

**FIGURE 9:**
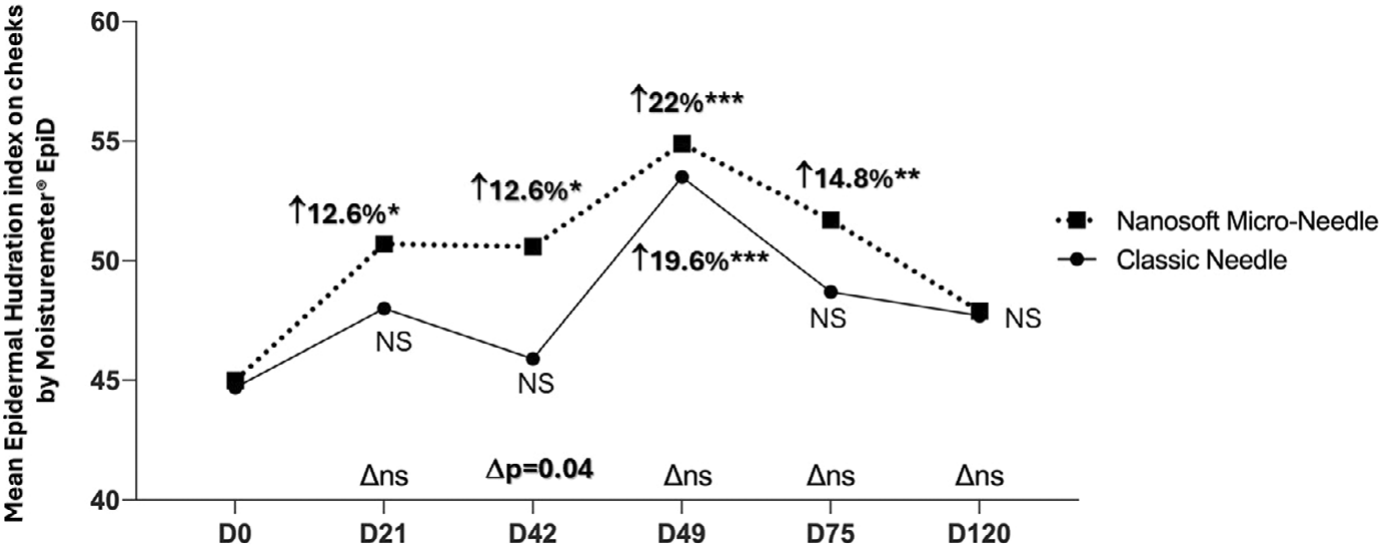
Mean epidermal hydration level (in percentage water content PWC %) measured by MoistureMeter EpiD assessed on the cheek. Significance value indicates *p<0.05, **p<0.01, ***p<0.001.

The face skin hydration level measured by MoistureMeter EpiD (Delfin technology, Finland) on cheeks was significantly improved on the Nanosoft side for all time points from D21 (3 weeks after only one injection session) until D75 (1 month after 3 injections) (D21 *p* = 0.02, D42 *p* = 0.01, D49 *p* < 0.0001, D75 *p* = 0.008) while the results are significant only 7 days after 3 injections (D49) for classic needle (*p* = 0.0002) (Table 4). Regarding the neck area, the side treated with Nanosoft showed a significant improvement of hydration compared to baseline at D21 (56.0 ± 4.1 vs. 53.5 ± 5.1 at D0; *p* = 0.01) and D49 (57.6 ± 6.4 vs. 53.5 ± 5.1 at D0; *p* < 0.006). However, the neck side treated with a classic needle only showed a better epidermal hydration level at D49 (55.4 ± 5.8 vs. 52.4 ± 4.6 at D0; *p* = 0.03) (Table 4). The difference between two devices is statistically significant for Nanosoft at D42 for the cheek zone (*p* = 0.04) (Figure 9) and at D75 for the neck area (*p* = 0.01).

**TABLE 4:** Biometrological parameters measured at all time points for Micro‐Needle or classic needle.

*n* = 19	Classic needle			Micro‐Needle			
	Mean	SD	*p*	Mean	SD	*p*	∆*p*
Deep hydration level of epidermis index							
Cheek zone							
D0	44.7	7.9	—	45.0	8.1	—	—
D21	48.0	9.0	ns	50.7	9.7	0.0218	ns
D42	45.9	8.0	ns	50.6	5.8	0.0135	0.0453
D49	53.5	8.1	0.0002	54.9	11.5	<0.0001	ns
D75	48.7	9.5	ns	51.7	11.8	0.0080	ns
D120	47.7	8.0	ns	47.9	9.1	ns	ns
Neck zone							
D0	52.4	4.6	—	53.5	5.1	—	—
D21	54.1	4.3	ns	56.0	4.1	0.0128	ns
D42	51.1	7.8	ns	52.3	6.6	ns	ns
D49	55.4	5.8	0.0329	57.6	6.4	0.0058	ns
D75	51.6	7.7	ns	55.3	6.5	ns	0.0166
D120	51.6	6.9	ns	52.2	6.8	ns	ns
SLEB index							
Cheek zone							
D0	46.0	89.0	—	56.0	104.0	—	—
D49	53.0	101.0	ns	49.0	94.0	ns	ns
D75	51.0	109.0	ns	60.0	119.0	ns	ns
D120	45.0	88.0	ns	47.0	107.0	ns	ns
Periorbital zone							
D0	151.6	127.5	—	130.7	118.8	—	—
D49	108.0	117.4	ns	110.5	134.6	ns	ns
D75	108.7	123.5	0.0427	111.2	113.2	ns	ns
D120	103.4	131	0.0499	116.6	120.3	ns	ns
Neck zone							
D0	70.7	86.2	—	81.3	93.7	—	—
D49	70.2	92.7	ns	53.0	73.2	0.0356	0.0191
D75	84.3	107.1	ns	63.3	89.1	ns	ns
D120	62.6	84.0	ns	69.2	84.7	ns	ns

Pain during injection score						
*n*=40	Classic needle			Micro‐Needle		
	Mean	SD	Median	Mean	SD	Median
Face zone						
D0	5.3	2.1	5.5	3.2	1.7	2.8
D21	5.1	2.2	5.1	3.3	2.3	2.4
D42	4.5	2.5	4.8	3.0	1.9	2.5
Neck zone						
D0	4.4	2.3	4.2	2.7	2.1	2.4
D21	4.8	2.4	4.9	3.0	2.3	2.3
D42	4.0	2.3	3.5	2.6	2.0	2.4

### 3.7 High‐Frequency Ultrasound Imaging

**FIGURE 10:**
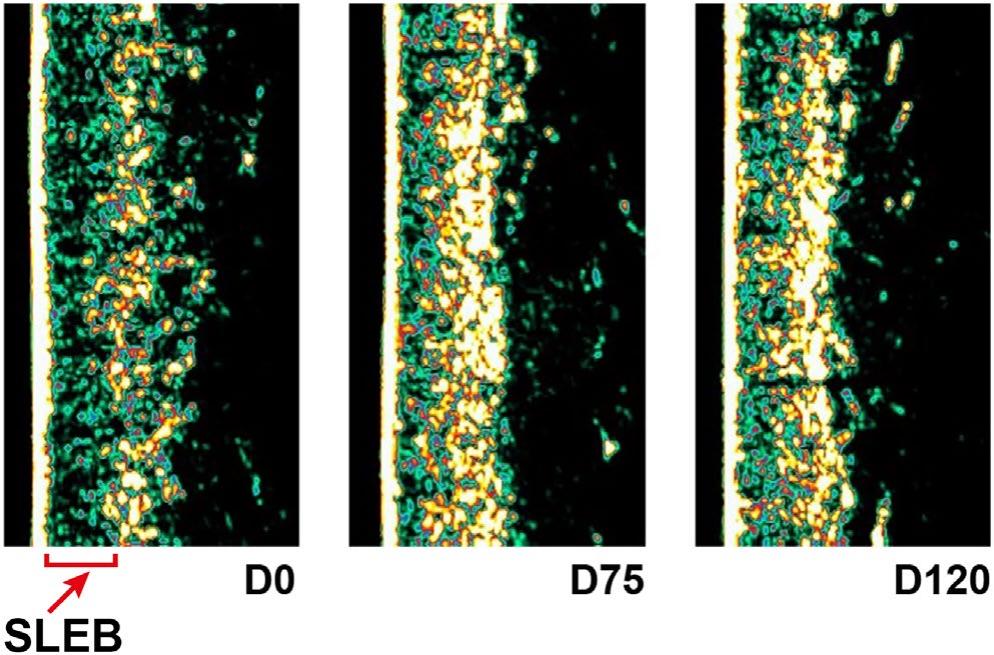
Visible decrease on SLEB from baseline to D75 and D120.

Moreover, at baseline (prior to injections), ultrasound showed the presence of SLEB (Subepidermal Low Echogenic Band) in all subjects, which is a reliable marker for skin photoaging grade (Figure 10).

#### 3.7.1 Regarding the Face Skin

The thickness measurements of SLEB highlighted few significant changes compared to baseline for both treated sides.

#### 3.7.2 Regarding the Neck Skin

A significant diminution of SLEB thickness was obtained for Micro‐Needle treatment at D49 (*p* < 0.04) (Table 4). In addition, there is a significant difference of evolution between treatments in favor to Micro‐Needle technology (*p* < 0.02).

### 3.8 Global Aesthetic Improvement Assessment

The satisfaction rate was evaluated by investigator GAIS (IGAIS) and also by subject GAIS (SGAIS) on two skin zones: neck and face (Figure S1.1 and S1.2).

#### 3.8.1 IGAIS

The investigator reported an excellent satisfaction rate for the face zone with 100% of improvement on both sides of the face at D42, D75, and D120. For the neck area, the same improvement rate on both sides were obtained at D42, D49, and D75.

#### 3.8.2 SGAIS

The patients observed an excellent improvement rate with a slight difference according to the mode of injections: 98% for the face zone from D49 to D120 with both classic needle and Micro‐needle, while with Micro‐Needle, the same percentage of improvement was achieved also at D42 (only after two injection sessions), which show that the results started sooner.

## 4 Safety and Tolerance

**FIGURE 11:**
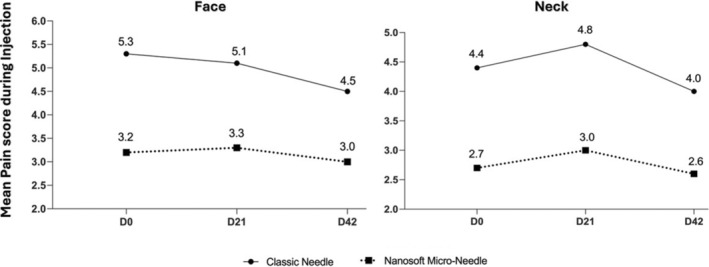
Pain during injection assessed on the face and the neck assessed after each injection session.

No serious adverse events have been reported during the study. Eighty‐one adverse events were reported by only 17% of the total population. Most of them were relating to expected adverse events that occurred frequently after dermal injections: erythema (*N* = 17/21%), burning sensations (*N* = 13/16%), irregularities on palpation (*N* = 10/13%), ecchymosis (*N* = 8/10%), pruritus (*N* = 2/3%) and pain (*N* = 1/1%). They did not last more than 11 days (mean duration of 3.1 ± 2.8 days with Classic needle and 2.4 ± 2.1 days with Micro‐needle). Pain reported during injection were lower with the Micro‐Needle than with the classic needle (about −2 on both mean and median values) for the face and the neck zone (Figure 11 and Table 4). Only one AE was not related to injection (COVID suspicion for one subject who stopped the study after the injection at D0).

## 5 Discussion

In this study, various measures were performed to re‐validate the performance of NCTF 135 HA and to evaluate any difference between the two injection modes: Classic needle versus Micro‐Needle. Antiaging biorevitalization can be indicated for tired or lack of radiance skin with intense dehydration [18], and the study data showed a significant improvement in radiance and wrinkles as early as 30 days after the last injection on both injected sides.

As discussed in the literature, biorevitalization is a mildly invasive procedure that involves subcutaneous drug injections to stimulate fibroblasts, increase collagen and elastin production, and improve skin properties [9, 10, 19]. Our previous study has shown that the use of intradermal microinjections of NCTF 135 HA in combination with biorevitalization have significant improvements in crow's‐feet wrinkles, pore size, dermatological scores, and skin tone [10].

Interestingly, Micro‐Needle injections appeared to be more efficient and rapid than classic needle injections for enhancing skin radiance. The same positive results were observed for skin wrinkles, with superior effects observed on the face for periorbital wrinkles, nasolabial fold and also for neck wrinkles. These findings were further supported by instrumental measures such as skin hydration level. High‐frequency ultrasound imaging provided deeper insights, revealing positive evolutions in the favor of the side treated by Micro‐Needle.

Considering that the skin thickness varies across different facial zones, and our results indicated measurable improvements primarily on cheeks and the neck. As the evaluation was conducted over a period of 4 months, it is possible that the periorbital area may require more time to fully demonstrate its restorative effects.

In terms of safety, our study found that the most adverse events encountered with both injection modes were quite similar. Notably, the pain reported during injection was significantly lower with the Micro‐Needle technology.

### 5.1 Conclusion

Overall, these findings highlight that NCTF 135 HA, particularly when administrated using Micro‐Needle injections, offers a compelling solution for antiaging biorevitalization. Micro‐Needle technology seems to be a more rapid, efficient, and safe device for biorevitalizing solutions. It is a suitable device to treat the most delicate zones such as periorbital area and neck. These results could be explained by the ex vivo studies which showed the remaining of the product on the right level until 24 h [9].

## Ethics Statement

The study adapted with the declaration the Helsinki with the authorization of the local ethical committee of Medical University of ‘Iuliu Haţieganu’ under registration number 2/1101.2019.

## Conflicts of Interest

F. F., H. I., N. S., and V. P. are the employee of FILLMED Laboratories in Paris. The other authors declare that they have no conflicts of interest in this work.

## Supporting information


**Figure S1.1.** Investigator and subject satisfaction rate (%) performed on face with two devices: Micro‐Needle versus classic needle evaluated by GAIS.

**Figure S1.2.** Investigator and subject satisfaction rate (%) performed on neck with two devices: Micro‐Needle versus classic needle evaluated by GAIS.

Table S1.

## Data Availability

The data that support the findings of this study are available on request from the corresponding author. The data are not publicly available due to privacy or ethical restrictions.
