# Supplementary figures and images for: Evaluation of the Performance and Safety of a New Micro‐Needle Technology in Comparison With the Classic Needle on the Antiaging Effects of a Biorevitalizing Solution: A Randomized Split Face/Neck Study
Source: J Cosmet Dermatol. 2024 Oct 2;23(12):3974–85. doi: 10.1111/jocd.16547 (PMC11626338; doi:10.1111/jocd.16547)

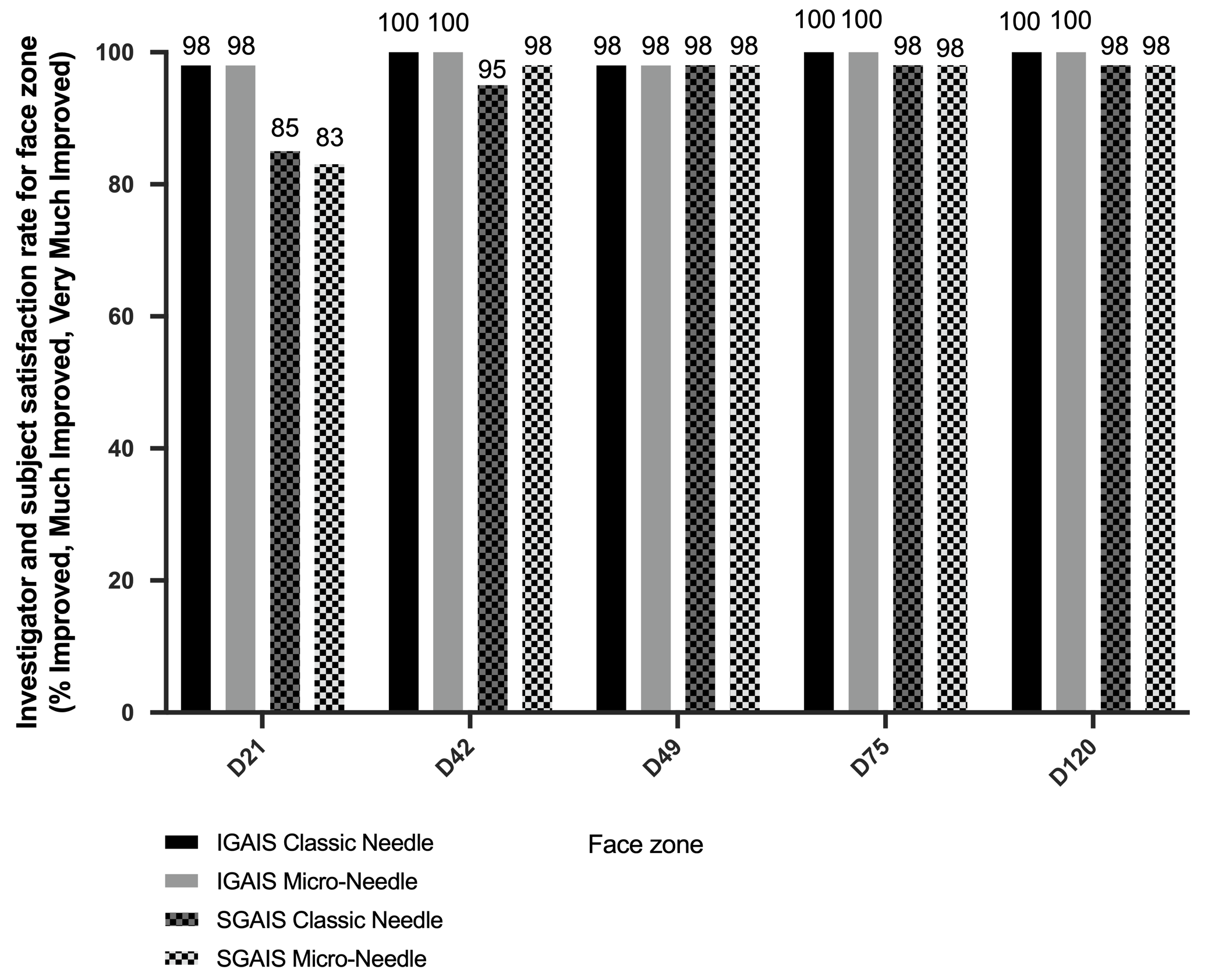

Supplement: Supplementary file 1 — Figure S1.1. Investigator and subject satisfaction rate (%) performed on face with two devices: Micro‐Needle versus classic needle evaluated by GAIS. [file JOCD-23--s002.tiff]

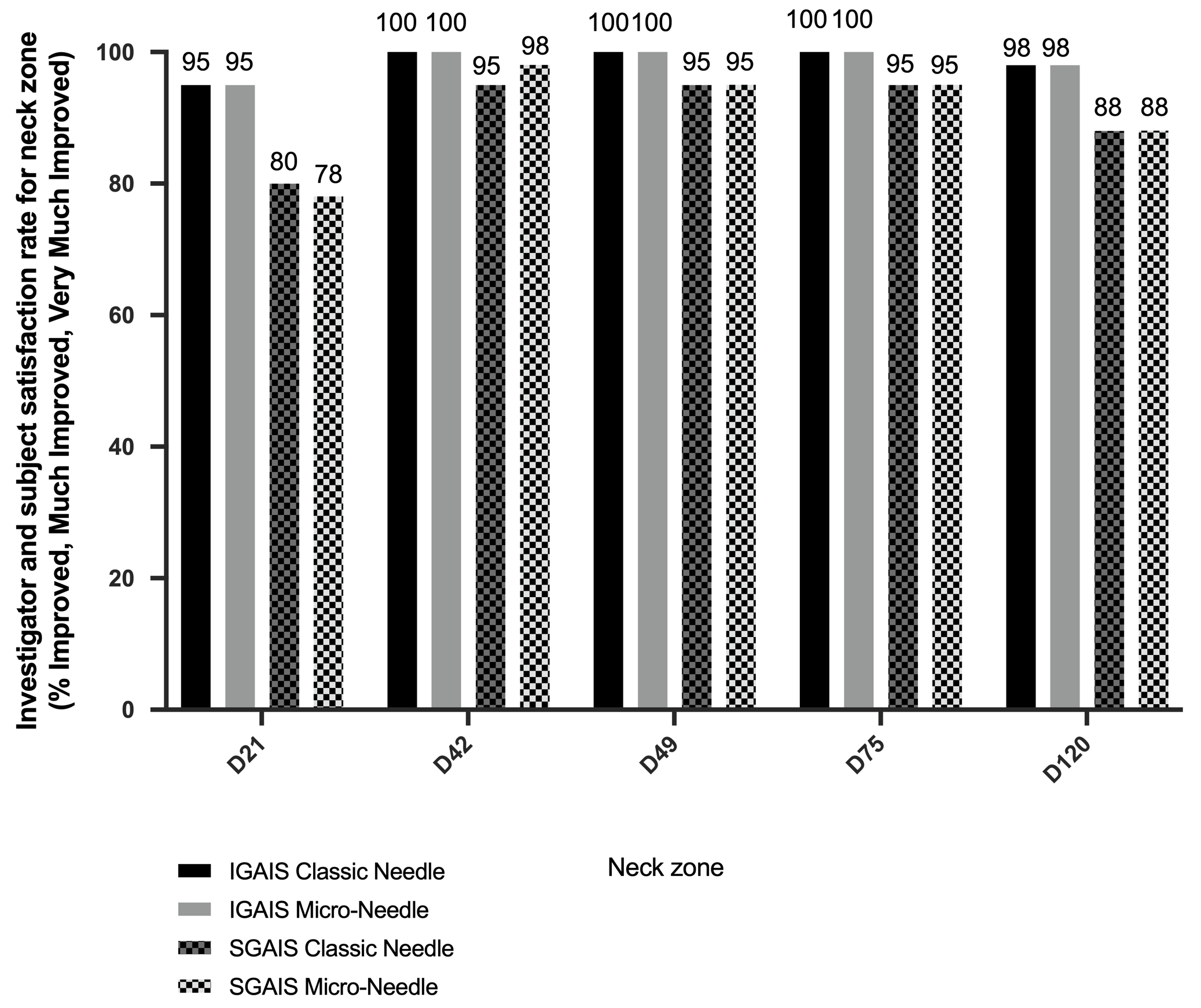

Supplement: Supplementary file 2 — Figure S1.2. Investigator and subject satisfaction rate (%) performed on neck with two devices: Micro‐Needle versus classic needle evaluated by GAIS. [file JOCD-23--s001.tiff]
