# Supplementary material for: Evaluation of the Performance and Safety of a New Micro‐Needle Technology in Comparison With the Classic Needle on the Antiaging Effects of a Biorevitalizing Solution: A Randomized Split Face/Neck Study
Source: J Cosmet Dermatol. 2024 Oct 2;23(12):3974–85. doi: 10.1111/jocd.16547 (PMC11626338; doi:10.1111/jocd.16547)
Supplement: Supplementary file 3 — Table S1. [file JOCD-23--s003.docx]

**Supplementary Tables**

**Table S1**

Additional non-invasive measuring techniques used to obtain objective evaluations of the treatment effects with two modes of injection: Micro-Needle or classic needle.

| **Dermis thickness** | | | | | | | | | |
| --- | --- | --- | --- | --- | --- | --- | --- | --- | --- |
| n=40 | **Classic needle** | | |  | **Micro-Needle** | | |  |  |
| **Cheek zone** | mean | SD | p |  | mean | SD | p |  | ∆p |
| D0 | 1405 | 269 | - |  | 1361 | 331 | - |  | - |
| D49 | 1345 | 314 | ns |  | 1426 | 304 | ns |  | p=0.0183 |
| D75 | 1392 | 226 | ns |  | 1468 | 311 | p=0.0171 |  | *p=0.0571* |
| D120 | 1440 | 280 | ns |  | 1418 | 297 | ns |  | ns |
| **Periorbital zone** |  | | |  |  | | |  |  |
| D0 | 1260 | 272 | - |  | 1242 | 295 | - |  | - |
| D49 | 1197 | 282 | ns |  | 1242 | 281 | ns |  | p=0.0420 |
| D75 | 1211 | 250 | ns |  | 1214 | 296 | ns |  | ns |
| D120 | 1252 | 298 | ns |  | 1215 | 296 | ns |  | ns |
| **Neck zone** |  | | |  |  | | |  |  |
| D0 | 1124 | 203 | - |  | 1134 | 204 | - |  | - |
| D49 | 1138 | 177 | ns |  | 1160 | 206 | p=0.0474 |  | ns |
| D75 | 1170 | 216 | p=0.0414 |  | 1189 | 218 | p=0.0022 |  | ns |
| D120 | 1111 | 218 | ns |  | 1141 | 188 | ns |  | ns |
|  |  |  |  |  |  |  |  |  |  |
| **VE index** | | | | | | | | | |
| n=40 | **Classic needle** | | |  | **Micro-Needle** | | |  |  |
| **Cheek zone** | mean | SD | p |  | mean | SD | p |  | ∆p |
| D0 | 0.37 | 0.20 | - |  | 0.32 | 0.22 | - |  | - |
| D49 | 0.34 | 0.17 | ns |  | 0.34 | 0.22 | ns |  | ns |
| D75 | 0.32 | 0.13 | ns |  | 0.29 | 0.17 | ns |  | ns |
| D120 | 0.40 | 0.13 | ns |  | 0.43 | 0.19 | p=0.0066 |  | p=0.03754 |
| **Periorbital zone** |  | | |  |  | | |  |  |
| D0 | 0.38 | 0.24 | - |  | 0.37 | 0.20 | - |  | - |
| D49 | 0.39 | 0.22 | ns |  | 0.40 | 0.23 | ns |  | ns |
| D75 | 0.43 | 0.30 | ns |  | 0.41 | 0.25 | ns |  | ns |
| D120 | 0.36 | 0.25 | ns |  | 0.43 | 0.28 | ns |  | p=0.0363 |
| **Neck zone** |  | | |  |  | | |  |  |
| D0 | 0.34 | 0.18 | - |  | 0.40 | 0.22 | - |  | - |
| D49 | 0.40 | 0.24 | ns |  | 0.36 | 0.23 | ns |  | ns |
| D75 | 0.34 | 0.19 | ns |  | 0.38 | 0.23 | ns |  | ns |
| D120 | 0.32 | 0.19 | ns |  | 0.34 | 0.22 | p=0.0451 |  | ns |
